# Supplementary material for: Doubling of surface oceanic meridional heat transport by non-symmetry of mesoscale eddies
Source: Nat Commun. 2023 Sep 6;14:5460. doi: 10.1038/s41467-023-41294-7 (PMC10482927; doi:10.1038/s41467-023-41294-7)
Supplement: Supplementary file 1 — Supplementary Information [file 41467_2023_41294_MOESM1_ESM.pdf]

Supplementary Information

Accompanying the article "Doubling of Surface Oceanic Meridional Heat Transport by Non-Symmetry of Mesoscale Eddies"

by

Hailin Wang[1], Bo Qiu[2], Hanrui Liu[1] & Zhengguang Zhang[1, 3,*]

[1]Frontiers Science Center for Deep Ocean Multispheres and Earth System (FDOMES) and Key Laboratory of Physical Oceanography, Academy of the Future Ocean, Chongben Honors College, Ocean University of China, Qingdao, P. R. China.
[2]Department of Oceanography, University of Hawaii at Manoa, Honolulu, Hawaii, USA.
[3]Laoshan Laboratory, Qingdao, P. R. China.

This Supplementary Information includes:

- Supplementary Notes 4
- Supplementary Table 1
- Supplementary Figures 1-9
- supplementary references

[*] Correspondence and requests for materials should be addressed to Z.Z. (zhengguang@ouc.edu.cn)

Supplementary Note 1 | Bootstrap test for the function fitting

A bootstrap test is conducted on the fitting factors used to reconstruct the directional index I_d to determine whether the contributions of each factor are significant[1-3]. For each factor, it global distribution data will be disordered randomly, at the same time, keeping the other variables unchanged, then the I_d is reconstructed accordingly and the correlations and relative errors are calculated. Repeated this process for 10,000 times to generate the random compare group. If the correlation coefficients and error are better than the 95% randomly generated correlation coefficients and errors, the contribution of the corresponding factor is significant. As shown in Table.S1, all variables pass the correlation coefficient test. The *p*-values of the bootstrap test for individual factors are computed, as shown in following Table.S1. If the *p*-value of a coefficient is smaller than 0.05, this term is significant at the 95% significance level. The *p*-values in Table.S1 are all well below this threshold, which indicates that all factors we adopted make significant contribution to the reconstruction. At the same time, the *p*-values for each term are all too small to determine the relative contribution of each term. Further *t*-test of the fitting is conducted and the results is also shown in Table.S1. For the *t*-test, if the *t*-statistic is larger than 2.571, the result would reach a 95% significant level in a linear fitting with five coefficients. The values in Table.S1 are also well above this threshold, and the planetary beta term has the largest *t*-statistic value.

Generally, all the factors used in the fitting equation have significant contributions to the reconstruction, and the planetary beta term may have the most important contribution.

Supplementary Note 2 | Eddy-induced heat flux changing with I_a

To evaluate the relation between eddy asymmetry and eddy-induced heat flux (EHF), the curves of I_a and total heat flux and its asymmetric/symmetric parts are computed. The non-axisymmetry index I_a from 1 to 3 is divided into 20 parts with a moving window size 0.4, calculating the corresponding total, asymmetric and symmetric meridional eddy induced heat flux EHF_y. The error bar indicates the standard error, defined as the standard deviation divided by the square root of the sample size. As shown in Fig.S1a, the meridional heat fluxes induced by the total eddy velocity field increase with the asymmetric index I_a. At same time, the meridional heat flux induced by the asymmetric/symmetric parts of the eddy flow field increases/decreases significantly with the increasing I_a, as shown in Fig.S1c and S1d. The contribution of the asymmetric part is nearly doubled (from 37% to 61%) with increasing I_a from 1 to 3, as shown in Fig.S1b. These results indicate that the elongated eddies will induce larger meridional heat flux due to the increasing contribution of its asymmetric part of the velocity field.

Supplementary Note 3 | Ratio of EHFs between symmetric/asymmetric eddies

In order to quantify eddy asymmetry's influence on meridional eddy-induced heat flux EHF_y in different parts of the world ocean, eddies are divided into two groups: asymmetric eddies with I_a greater than 1.55, and the nearly-symmetric eddies eddies with I_a less than 1.55 (the average value of I_a is 1.55). Calculate the global EHF_y distribution on a $2° \times 2°$ grid with a longitude 8 degrees and latitude 6 degrees' window for the two groups. Divide the global EHF_y distribution from eddies with I_a greater than 1.55 by the global EHF_y distribution from eddies with I_a less than 1.55, resulting in the ratio shown in Fig.S2. As shown in Fig.S2, the asymmetric eddies tend to hinder the meridional heat flux in low latitudes where the ratio is smaller than one and the heat flux is almost doubled in the high latitude regions by the eddy asymmetry. In a globally average sense, the asymmetry increases the eddy-induced meridional heat flux by about 20%. In mid-high latitude regions, the asymmetry increases the eddy-induced meridional heat flux by about 50%-100%.

Supplementary Note 4 | Eddy Asymmetry Changes During Its Lifespan

Eddy asymmetry could change during its whole lifespan. In order to obtain the change rate, the average I_a change curve is calculated. Different eddy has different length of life. Thus, the I_a of eddy with different length of life is normalized into same length of life from 0 to 1 through interpolation. Then take the average of all the I_a change curves normalized by eddy lifespan to obtain the mean curve shown in Fig.3. During the growing and decaying stage, Eddy has the weakest symmetry, corresponding to a relatively large I_a. When eddy become stable, eddy has small I_a and high symmetry.

Table.S1 | The first row shows the factors used to reconstruct the global distribution of directional index. The second to six rows are the bootstrap test results, using correlation coefficient (Corr. Coeff.) and relative error (Rel. Err.) to test whether the factors have significant contributions to the reconstruction. If the correlation coefficient/relative error of the tested term is larger/smaller than the 95% of the random group outputs, contribution of the corresponding factor is significant. The *p*-value in the six row is computed by one-sided empirical method. The seven row is the results of *t*-test for the linear fitting. *T*-statistic for each coefficient tests the null hypothesis that the corresponding coefficient is zero against the alternative that it is different from zero, given the other predictors in the model.

Fitting Effect	$\boldsymbol{\beta_{bx}}$	$\boldsymbol{\beta_{by}}$	$\boldsymbol{\beta_{gx}}$	$\boldsymbol{\beta_{gy}}$	$\boldsymbol{f(\beta)}$
Random 95% Corr. Coeff.	0.7131	0.7172	0.7061	0.7141	0.4363
Tested Term Corr. Coeff.	0.7295	0.7295	0.7295	0.7295	0.7295
Random 95% Rel. Err.	37.15%	36.25%	37.86%	36.32%	55.18%
Tested Term Rel. Err.	36.47%	36.47%	36.47%	36.47%	36.47%
p-value	$<10^{-4}$	$<10^{-4}$	$<10^{-4}$	$<10^{-4}$	$<10^{-4}$
t-statistic	40.925	35.501	40.865	40.076	172.42

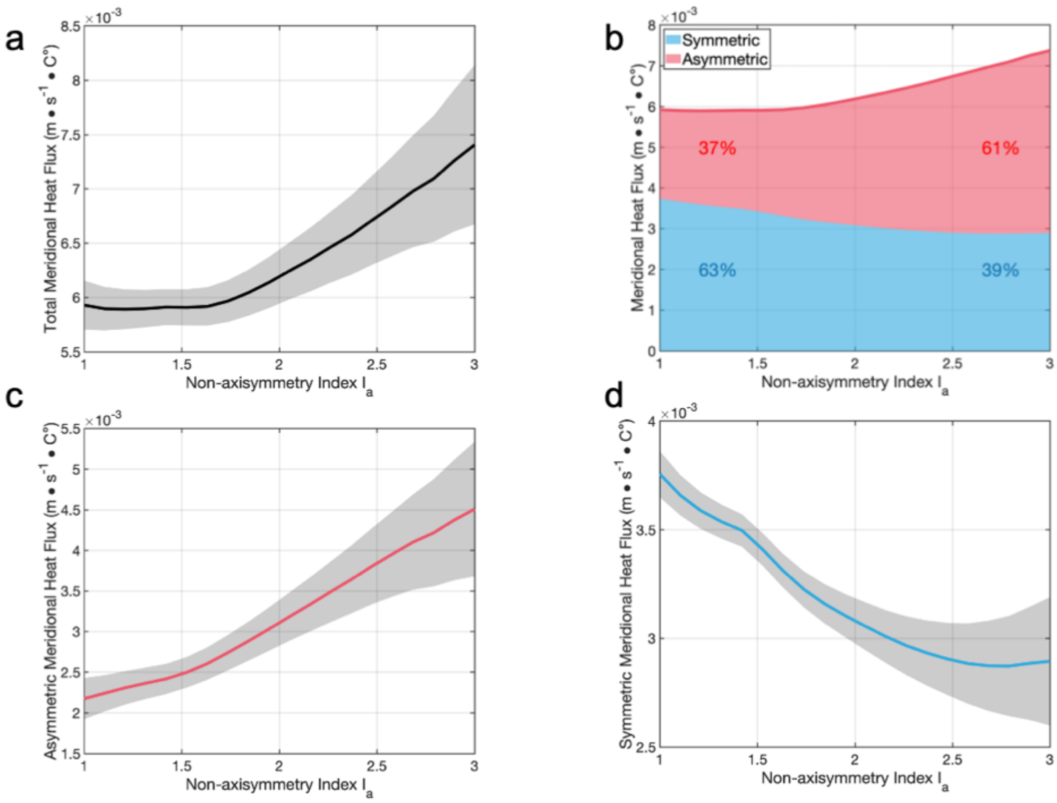

Figure.S1 | (a) Curve of the total meridional eddy-induced heat fluxes (EHF) changing with the eddy asymmetric index I_a. **(b)** The proportions of asymmetric and symmetric parts of the total EHF changing with I_a. The red area represents the asymmetric part and the blue area for the symmetric part. The percentage numbers on the left correspond to $I_a = 1$ (axis-symmetric eddy), while the percentage numbers on the right correspond to $I_a = 3$ (extremely elongated eddy). **(c)** Curve of the meridional EHF induced by asymmetric velocity field changing with I_a. **(d)** same as (c) but for symmetric part. The gray range represents the error bar in (a)(b)(c), which is calculated as the standard error. The asymmetric index $I_a = a/b$ is introduced as the ratio between the major and minor axis of eddies. Source data are provided as a Source Data file.

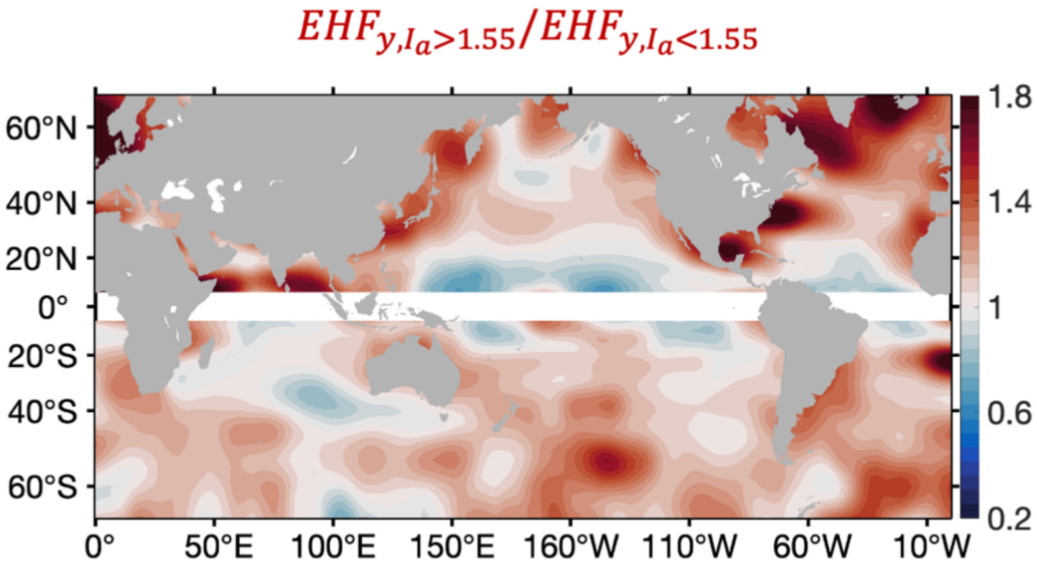

Figure.S2 | Global distribution for the ratio of total meridional eddy-induced heat flux EHF_y with I_a greater than 1.55 and less than 1.55. Source data are provided as a Source Data file.

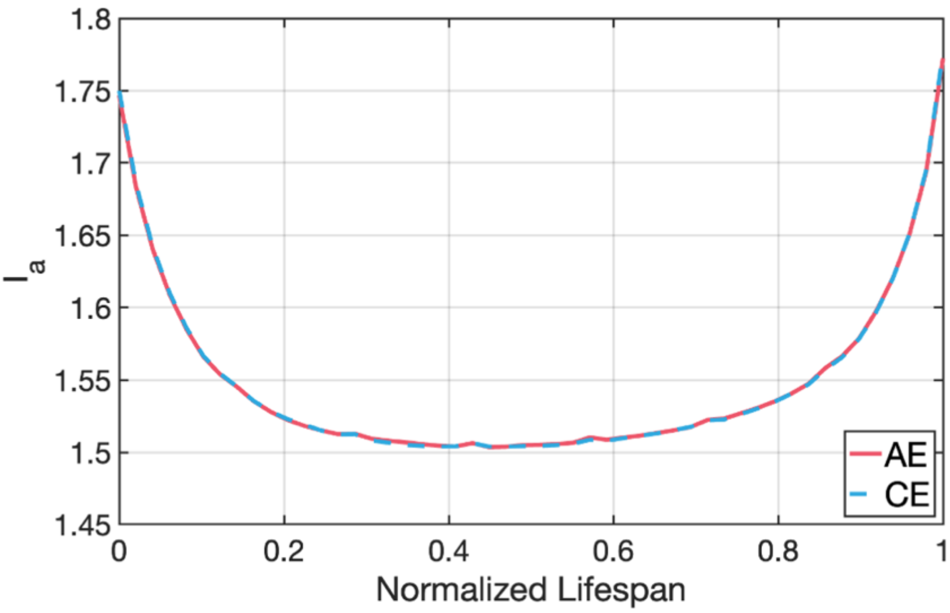

Figure.S3 | The average I_a change during eddy's lifespan. The red solid line represents anticyclonic eddy and the blue dashed line represents cyclonic eddy. The x-axis is the normalized lifespan, which interpolate different length of eddy life into the same length. Source data are provided as a Source Data file.

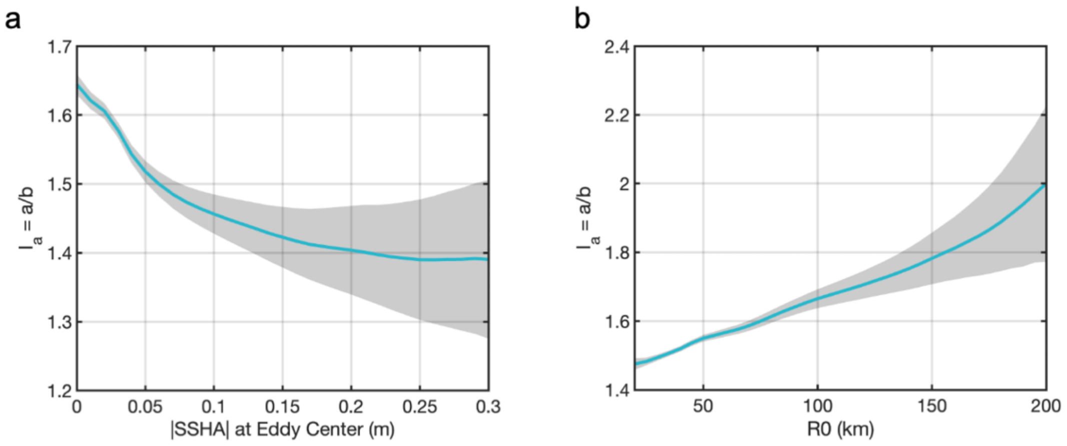

Figure.S4 | (a) Globally-averaged curve of index I_a as a function of the eddy amplitude (|SSHA| at eddy center). **(b)** Globally-averaged curve of index I_a as a function of eddy radius (R_0). Blue curve in each subfigure represents the average value and gray shading represents the error bar computed by the standard error of average. Source data are provided as a Source Data file.

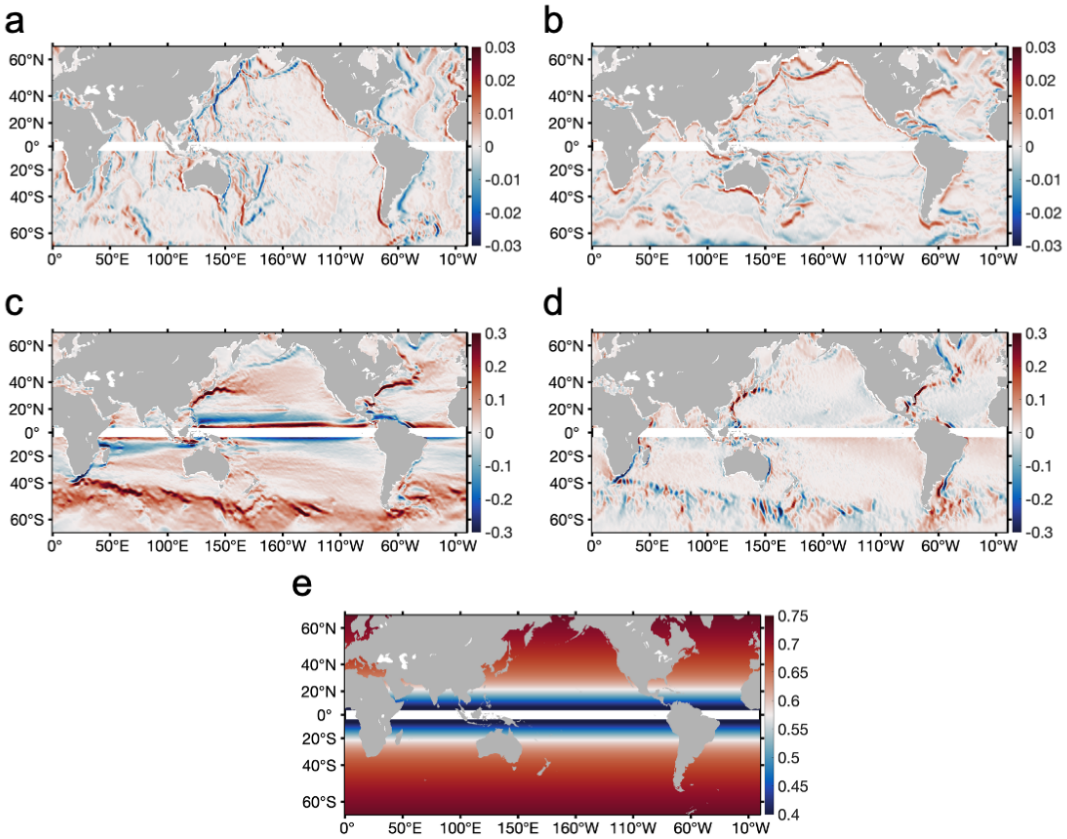

Figure.S5 | Factors used to fit the global I_d distribution. **(a)** and **(b)** are the global distribution of the zonal and meridional topography gradient, respectively. **(c)** and **(d)** are the global distribution of the multi-year average zonal and meridional surface geostrophic current, respectively. **(e)** Global distribution of the $f(\beta)$. Before the reconstruction of I_d, all the factors will be standardized (subtract average and divided by standard deviation). Source data are provided as a Source Data file.

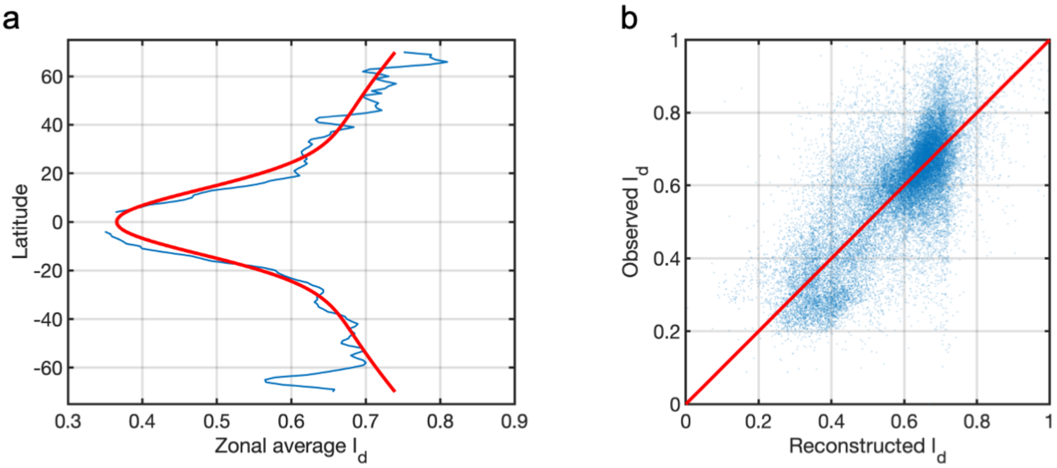

Figure.S6 | (a) The zonal averaged distribution of eddy directional index. The blue line is the observed zonal averaged values of the I_d, and the red line is the fitted curve using planetary beta effect given by equation (4) in the main text. **(b)** The scatter compared the observed and reconstructed global distribution of eddy directional index I_d. Source data are provided as a Source Data file.

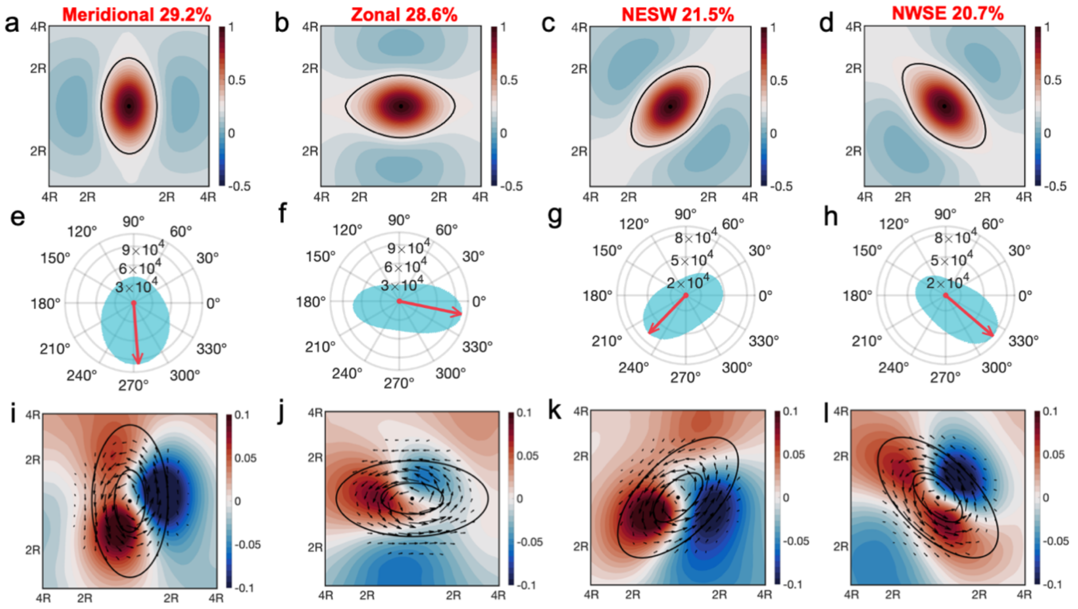

Figure.S7 | Direction of eddy-induced heat flux controlled by eddy directional-dependence in Southern Hemisphere observed by satellite. (a)-(d) Spatial structures of composited normalized sea surface height anomaly (SSHA) distributions in the normalized eddy center coordinate of (a) meridional, (b) zonal, (c) Northeast-Southwest (NESW) and (d) Northwest-Southeast (NWSE) directed eddies, respectively. Color represents the normalized SSHA and black bold contours represent eddies' boundary. Red number over each subfigure represents the percentage of eddies with the corresponding direction among all observed eddies. **(e)-(h)** Eddy-induced surface heat flux directions by the (e) meridional, (f) zonal, (g) NESW and (h) NWSE directed eddies in the Southern Hemisphere. Blue shade is the eddy counting numbers for each direction and red vector represents the averaged direction of the eddy-induced heat flux of all available eddies with the corresponding direction. **(i)-(l)** are the corresponding sea surface temperature and surface velocity anomaly fields of anticyclonic eddies in the Southern Hemisphere, both composited by satellite remote sensing data. Black vectors represent the surface velocity anomaly field, and color shade represents the sea surface temperature anomalies. Black contours in each subfigure represent the ideal eddy boundary. Source data are provided as a Source Data file.

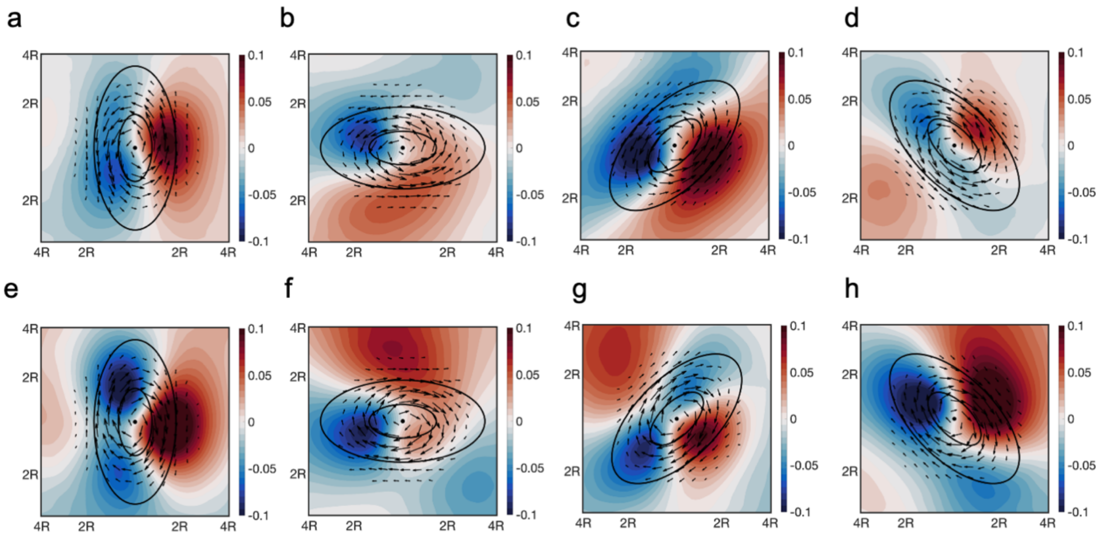

Figure.S8 | Composited sea surface temperature and surface velocity anomaly structure of cyclonic eddies in both hemispheres observed by satellite. (a)-(d) Composited sea surface temperature and surface velocity anomaly fields of cyclonic eddies in the Northern Hemisphere of (a) meridional, (b) zonal, (c) Northeast-Southwest (NESW) and (d) Northwest-Southeast (NWSE) directed eddies. **(e)-(h)** are the same as (a)-(d), but for cyclonic eddies in the Southern Hemisphere. All of the results are composited by satellite remote sensing data. Black vectors represent the surface velocity anomaly field, and color shade represents the SST anomalies. Black contours in each subfigure represent the ideal eddy boundary. Source data are provided as a Source Data file.

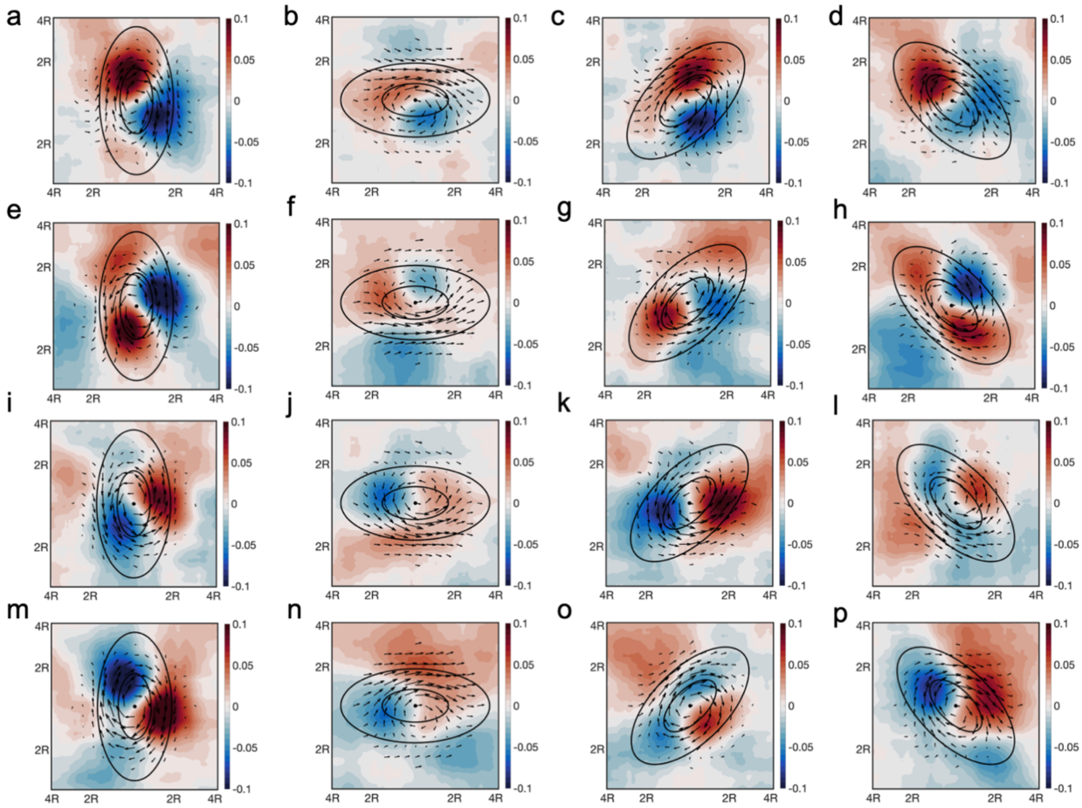

Figure.S9 | Composited sea surface temperature and surface velocity anomaly structure observed by surface drifters. (a)-(d) sea surface temperature and surface velocity anomaly fields of anticyclonic eddies in the northern hemisphere for four eddy directions: (a) meridional, (b) zonal, (c) Northeast-Southwest (NESW) and (d) Northwest-Southeast (NWSE) directed eddies. **(e)-(h)**, **(i)-(l)** and **(m)-(p)** are the same as (a)-(d), but for anticyclonic eddies in the south hemisphere, cyclonic eddies in the northern hemisphere and cyclonic eddies in the south hemisphere. Black vectors represent the surface velocity anomaly field, and color shade represents the sea surface temperature anomalies. Black contours in each subfigure represent the ideal eddy boundary. All of the results are composited based on drifter data. Source data are provided as a Source Data file.

supplementary references

1. Stine, R. An introduction to bootstrap methods: Examples and ideas. *Sociol. Methods Res.*, **18(2-3)**, 243-291 (1989).
2. Hall, P., & Hart, J. D. Bootstrap test for difference between means in nonparametric regression. *J. Am. Stat. Assoc.*, **85(412)**, 1039-1049 (1990).
3. Li, Q., & Wang, S. A simple consistent bootstrap test for a parametric regression function. *J. Econom.*, **87(1)**, 145-165 (1998).
